# Screening of M^pro^ Protease (SARS-CoV-2) Covalent Inhibitors from an Anthocyanin-Rich Blueberry Extract Using an HRMS-Based Analytical Platform

**DOI:** 10.3390/molecules29112702

**Published:** 2024-06-06

**Authors:** Alessandra Altomare, Giovanna Baron, Giulia Cambiaghi, Giulio Ferrario, Beatrice Zoanni, Larissa Della Vedova, Giulio Maria Fumagalli, Sarah D’Alessandro, Silvia Parapini, Serena Vittorio, Giulio Vistoli, Patrizia Riso, Marina Carini, Serena Delbue, Giancarlo Aldini

**Affiliations:** 1 Department of Pharmaceutical Sciences (DISFARM), Università degli Studi di Milano, Via Mangiagalli 25, 20133 Milan, Italy; giovanna.baron@unimi.it (G.B.); giulia.cambiaghi@icloud.com (G.C.); giulio.ferrario1@unimi.it (G.F.); beatrice.zoanni@unimi.it (B.Z.); larissa.dellavedova@unimi.it (L.D.V.); serena.vittorio@unimi.it (S.V.); giulio.vistoli@unimi.it (G.V.); marina.carini@unimi.it (M.C.); giancarlo.aldini@unimi.it (G.A.); 2 Unitech OMICs, Università degli Studi di Milano, Viale Ortles 22/4, 20139 Milan, Italy; giulio.fumagalli@unimi.it; 3 Department of Pharmacological and Biomolecular Sciences, Università degli Studi di Milano, Via Carlo Pascal 36, 20133 Milan, Italy; sarah.dalessandro@unimi.it; 4 Department of Biomedical Sciences for Health, Università degli Studi di Milano, Via Carlo Pascal 36, 20133 Milan, Italy; silvia.parapini@unimi.it; 5 Department of Food, Environmental and Nutritional Sciences, Università degli Studi di Milano, Via Luigi Mangiagalli 25, 20133 Milan, Italy; patrizia.riso@unimi.it; 6 Department of Biomedical, Surgical and Dental Sciences, Università degli Studi di Milano, Via Carlo Pascal 36, 20133 Milan, Italy; serena.delbue@unimi.it

**Keywords:** metabolomics, mass spectrometry, SARS-CoV-2, M^pro^, blueberry, delphinidin-3-glucoside

## Abstract

Background: The viral main protease (M^pro^) of SARS-CoV-2 has been recently proposed as a key target to inhibit virus replication in the host. Therefore, molecules that can bind the catalytic site of M^pro^ could be considered as potential drug candidates in the treatment of SARS-CoV-2 infections. Here we proposed the application of a state-of-the-art analytical platform which combines metabolomics and protein structure analysis to fish-out potential active compounds deriving from a natural matrix, i.e., a blueberry extract. Methods: The experiments focus on finding MS covalent inhibitors of M^pro^ that contain in their structure a catechol/pyrogallol moiety capable of binding to the nucleophilic amino acids of the enzyme’s catalytic site. Results: Among the potential candidates identified, the delphinidin-3-glucoside showed the most promising results. Its antiviral activity has been confirmed in vitro on Vero E6 cells infected with SARS-CoV-2, showing a dose-dependent inhibitory effect almost comparable to the known M^pro^ inhibitor baicalin. The interaction of delphinidin-3-glucoside with the M^pro^ pocket observed was also evaluated by computational studies. Conclusions: The HRMS analytical platform described proved to be effective in identifying compounds that covalently bind M^pro^ and are active in the inhibition of SARS-CoV-2 replication, such as delphinidin-3-glucoside.

## 1. Introduction

The COVID-19 pandemic stalled the world, and the massive vaccination campaign, hopefully leading to herd immunity, is the only weapon we have to fight it today. However, SARS-CoV-2 is a virus that is particularly prone to mutations [1] and although the vast majority of variants will not arouse any interest, some may prove capable of evading vaccine-induced immunity. While booster vaccine doses could be developed for new variants, small bioactive molecules as antivirals, designed against less mutable intracellular targets, would be more successful than a vaccine in this battle [2]. Hence, the development of effective interventions against SARS-CoV-2 continues to be a top priority; however, it must be considered that the development of a specific therapy could take months or years [3]. Looking at the replicative cycle of SARS-CoV-2, there are several critical steps that, if inhibited, would compromise viral replication, and thus many viral targets should be investigated. In this regard, the 3-chymotrypsin-like protease, or main protease (3CL^pro^ or M^pro^ [4]), has been considered one of the main druggable targets since the beginning of COVID-19’s emergence. M^pro^ is a key enzyme mediating SARS-CoV-2 replication and spread, by digesting the two viral polyproteins into vital non-structural proteins (nsp) [5,6,7,8]. To date, extensive research has led to the identification of potential ‘inactivators’ of the M^pro^ enzyme.

The first strategy pursued was ‘drug repurposing’, whereby broad-spectrum antiviral agents, previously tested for other viral infections, were tested to assess their efficacy against SARS-CoV-2. The second approach for developing antivirals, currently the most widely used, involves virtual screening campaigns and High Throughput Screening (HTS) assays based on chemical libraries of known compounds; this in silico search for molecules within a database of interest is actually limited to the current know-how, thus reducing the exploratory power. Nevertheless, this approach allowed the definition of specific ‘chemotypes’ with high affinity for the M^pro^ binding site, mainly occurring in natural compounds, further underlining the enormous potential of plants as a natural source of bioactive molecules due to their marked structural heterogeneity, the broad range of beneficial effects promoted and their reduced toxicity compared to synthetic drugs [9,10,11].

A third path, one characterized by the greatest experimental complexity, is the de novo design and development of viral replication process inhibitors. Ideally, this approach would lead to the development of molecules characterized by considerable potency. However, these processes are very long and costly, and require the fulfilment of complex legislative procedures in terms of drug approval [4].

Against this background, screening strategies aimed at identifying bioactive natural compounds as potential M^pro^ inhibitors could have a substantial impact in the fight against SARS-CoV-2. In this scenario, we proposed an innovative strategy, based on high-resolution mass spectrometry (HR-MS) and integrating advanced methods for metabolomics and protein structure analysis to elucidate the identity and the binding mode of covalent M^pro^ binders [12]. Furthermore, thanks to the state-of-the-art equipment used, which enables high-sensitivity analytical performance levels, it has been possible to work in untargeted mode, thus identifying any unknown or non-commercially available compounds by testing a wide range of natural extracts and expanding the current knowledge base. Briefly, the sophisticated HRMS-based platform was designed for: (i) qualitative/semi-quantitative characterization of natural extracts (LC-HR-MS/MS); (ii) in vitro screening of electrophilic compounds capable of forming Michael adducts with a thiol group (cysteine); and (iii) M^pro^ covalent binders and corresponding affected nucleophilic site identification (protein structure analysis; bottom-up approach). 

In this work, a blueberry extract rich in anthocyanins was chosen for screening and testing of its components using the HRMS-based-platform here cited; the choice was driven by the presence in the mixture of such moieties attributable to a potential reactivity towards residues constituting the M^pro^ active site [13].

## 2. Results

### 2.1. Metabolomics

#### 2.1.1. Analytical Profiling of BPE

The analytical profile of BPE was resolved by LC-HR-MS/MS analysis (positive ion mode). The chromatogram identified 18 well-solved species eluting within 45 min (Figure S1, Supplementary Materials), the peaks of which, for convenience, have been sequentially numbered according to their elution time.

Based on the information contained in the database, it was possible to characterize the extract’s compounds. Specifically, the compounds were annotated by extracting the ionic current of the *m*/*z* value (calculated as [M]^+^ or [M + H]^+^) with a tolerance of 5 ppm, and comparing the experimental isotopic and fragmentation patterns with the theoretical ones. The list of the 18 identified constituents is shown in Table 1 according to the elution order. 

The first peaks correspond to more hydrophilic molecules (e.g., anthocyanins), while as retention time increases, so does the compound’s lipophilicity. Of all the components known to occur in blueberry, in line with the literature, anthocyanins were found to be the predominant class [14]. A clarification should be made here, which will be valid for all investigations conducted from this point onwards: the glycosidic forms of the flavonoids under investigation, specifically those conjugated with glucose and galactose units, are not distinguishable in MS since they are isobaric species; moreover, with such similar chemical-physical characteristics they co-elute in chromatography.

**Figure 1:**
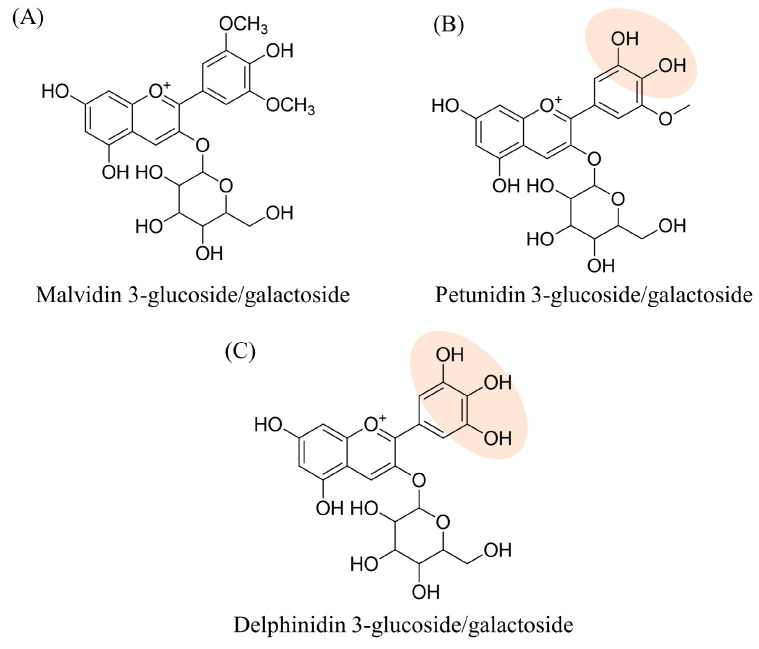
Structure formulae of the three most abundant components of BPE, in two of which the catechol/pyrogallol portion is highlighted.

Along with the qualitative definition of the natural extract, for each compound, its relative abundance was estimated by means of a semi-quantitative profile reworking. This estimation was accomplished by automatically integrating the area under the curve (AUC) of the extracted chromatographic peak for each compound, for which the percentage contribution to the total was calculated. Relative abundance values are shown in Table 1 using a color code: starting with the lowest value highlighted in dark green, moving through white and finally to red for the highest values. A prevalence of the glycosidic form of malvidin, petunidin and delphinidin emerges very clearly (Figure 1).

**Figure 2:**
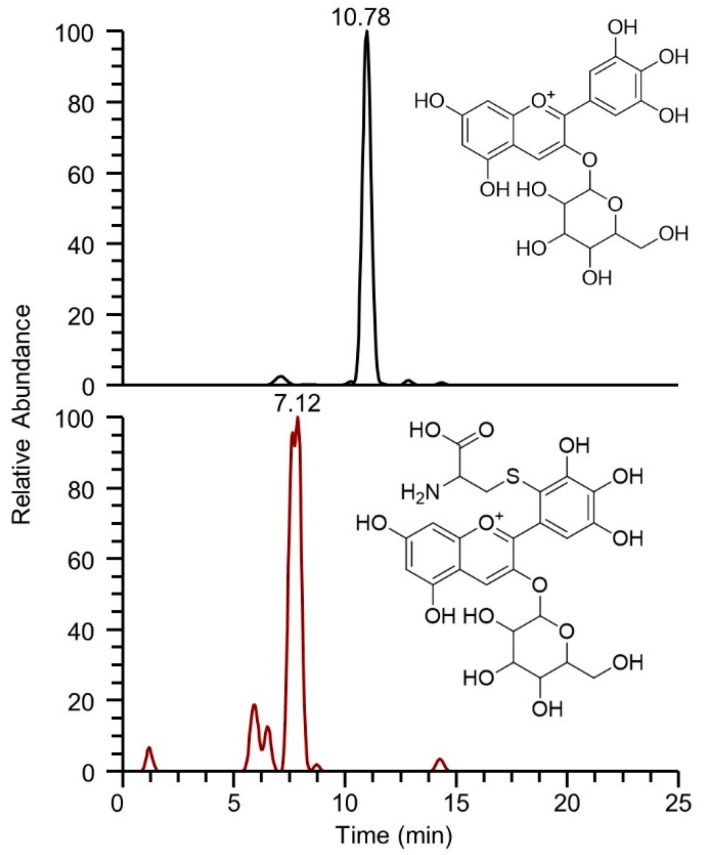
Extracted ionic currents for delphinidin-3-glucoside/galactoside having *m*/*z* 465.1033 (**upper** panel), and for the corresponding delphinidin-3-glucoside/galactoside-Cys adduct having *m*/*z* 584.10740 (**lower** panel), obtained from the re-elaboration of the chromatogram acquired at the 2 h time point.

Although the three flavonoid derivatives in Figure 2 share the resorcinol moiety susceptible to Michael addition, this type of reactivity is generally not observed for the resorcinol ring of anthocyanins. Indeed, several reports indicate that, in solutions, the 6 and 8 positions of anthocyanins have a nucleophilic character rather than electrophilic properties [15,16]. In addition, the literature suggests that flavonoid derivatives bearing both the resorcinol and the pyrogallol rings are more likely to undergo Michael addition on the latter. Some examples include myricetin, which forms a covalent adduct with Cys145 of M^pro^ through the pyrogallol moiety [17], as well as EGCG, which reacts with the glutathione sulfhydryl group at the C2′ or C6′ of the same ring [18,19]. Based on these findings, we hypothesized that the pyrogallol moiety is more prone to react with the nucleophilic residues of M^pro^ catalytic dyad. Among the three most abundant components of BPE, two share the presence of a catechol/pyrogallol moiety, i.e., a di/tri-hydroxylated ring that oxidizes to quinone becoming more susceptible to nucleophilic attack via the Michael addition mechanism. Another relevant consideration is the accuracy of the recorded experimental mass values compared to the theoretical ones; the calculated ∆ppm values fall within the assumed tolerance range with an average value of −1.687. Finally, it was useful at this stage to annotate the retention time and characteristic fragments for each compound, information that will be helpful in subsequent analyses.

#### 2.1.2. Identification of BPE Electrophilic Compounds and Reaction Kinetics Study

Once the full profiling of the extract was accomplished, the focus was on identifying those electrophilic compounds capable of reacting with thiolate, a reaction that represents the basic mechanism of many M^pro^ inhibitors acting as covalent ligands at the binding site. Cysteine was used as a thiol model and its incubation with the extract was carried out at different time points as reported in the methodological section. The formulae of BPE compound adducts with Cys were hypothesized, the exact mass was calculated (Table 2) and the corresponding ion current was extracted from the traces acquired at specific time points.

At this point, isorhamnetin-3-glucoside/galactoside, malvidin-3-arabinoside, malvidin-3-glucoside/galactoside, peonidin-3-arabinoside and peonidin-3-glucoside/galactoside were excluded from the panel of electrophilic compounds since their structural formulae do not contain an available catechol moiety that promotes the formation of the highly reactive quinone intermediate by oxidative activation; this is the basic mechanism through which many catechol-containing natural products react with M^pro^, as recently demonstrated [12]. All the hypothesized structural formulae for the covalent adducts resulting from the reaction of each phytocomponent with cysteine were confirmed, except for petunidin-3-glucoside/galactoside, which exhibits a reduced reactivity due to O-methylation on the 3-hydroxy group of pyrogallol (methylated derivative of delphinidin-3-glucoside/galactoside).

As an example, Figure 2 shows the extracted ion currents relative to delphinidin-3-glucoside/galactoside and delphinidin-3-glucoside/galactoside-Cys adduct. The presence of both species confirms the reactivity of the pyrogallol moiety characteristic of delphinidin glycoside towards the thiol group of cysteine. Another plausible consideration is the increased hydrophilicity of the adduct, which results in an earlier elution compared to the starting compound, with a retention time shift of about 3.5 min. 

**Figure 3:**
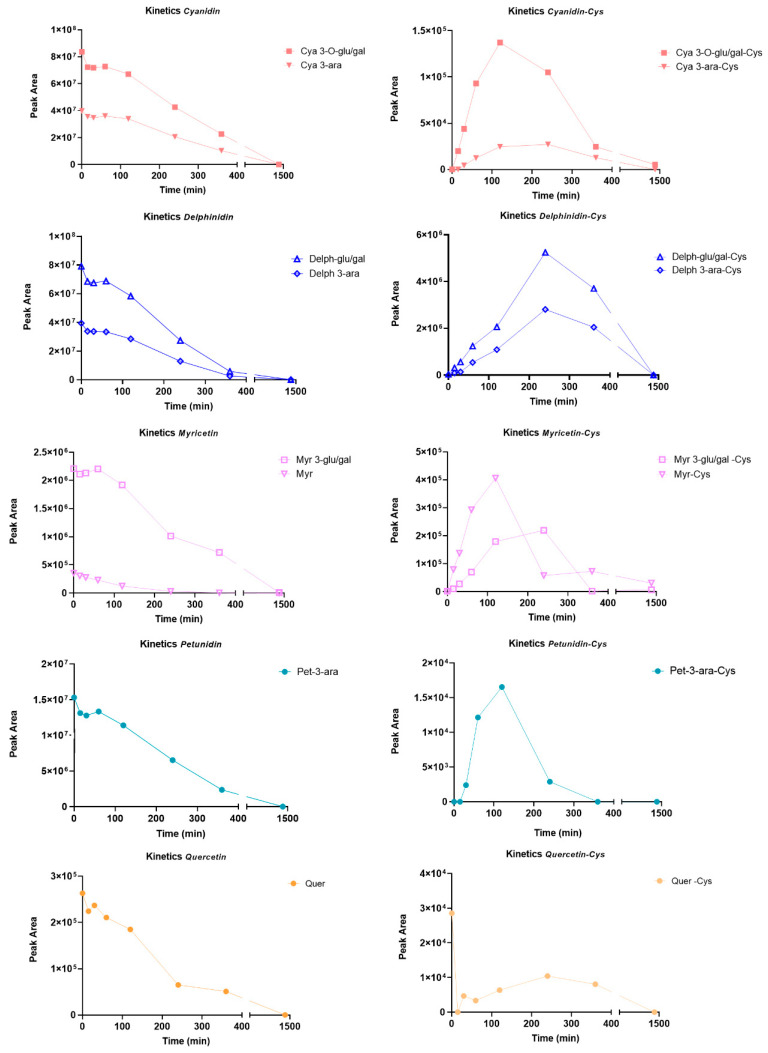
Time-dependent peak areas of BPE’s compounds (**left** panels) and Cys-BPE’s compound adducts (**right** panels) at different time points.

A key point, which we have partly anticipated, concerns the reactivity of flavonoids, which in the di/tri-hydroxylated form are little or non-reactive; we therefore have to assume a quinone oxidation process that anticipates nucleophilic attack. It has already been reported in the literature that flavonoids undergo a process of autoxidation in the presence of oxygen [20]; quinones are more susceptible to nucleophilic attack via Michael adduction or through the formation of a Schiff base by the side chains of nucleophilic residues. In order to follow the kinetics of adduct formation, affected by the flavonoids’ reactivity in the mixture towards the cysteine residue, an incubation test of the extract with cysteine was performed under physiological conditions in which the only oxidizing agent was oxygen from the air. The kinetics were followed over a 24 h time interval, at different set sampling times, and the parameter that allowed evaluation of the trend of single molecule content and relative adducts formed over time was still the AUC value calculated for each species (Figure 3). However, this approach has limitations due to the impossibility of relating the recorded ion current to the physical and chemical properties of each component, which significantly affect ionization efficiency.

All kinetic curves share a time-dependent trend: a gradual decrease in the relative content of the constituents corresponds to an increasing development of adducts, with the maximum reached between 2 and 4 h of incubation; after this time, it was assumed that the observed drop in the compound relative content may be due to degradation or rearrangement phenomena, resulting in the formation of species which were not detectable in the analysis conditions.

In order to speculate on a ranking of reactivity towards the cysteine residue, the absolute AUC values of the adducts formed in the presence of the targeted molecules were examined:

(i) Among the adducts formed in the presence of the glycosidic derivatives of the target flavonoids, the delphinidin-3-glucoside/galactoside-Cys adduct is definitely the predominant one, with a significantly higher content than the adducts formed in the presence of the glycosylated forms of myricetin, cyanidin and petunidin (Delph > Myr > Cya > Pet);
(ii) In general, adducts with derivatives carrying the glucose/galactose units bound are more abundant than those formed with molecules carrying arabinose units;
(iii) The last plausible consideration concerns the different reactivity observed between the glycosidic forms and the corresponding aglycones; actually, the only flavonoid present in both forms is the myricetin. The glycosidic derivative has a better Tmax and a content which is twice as high as the adduct produced with the corresponding aglycone.

**Figure 4:**
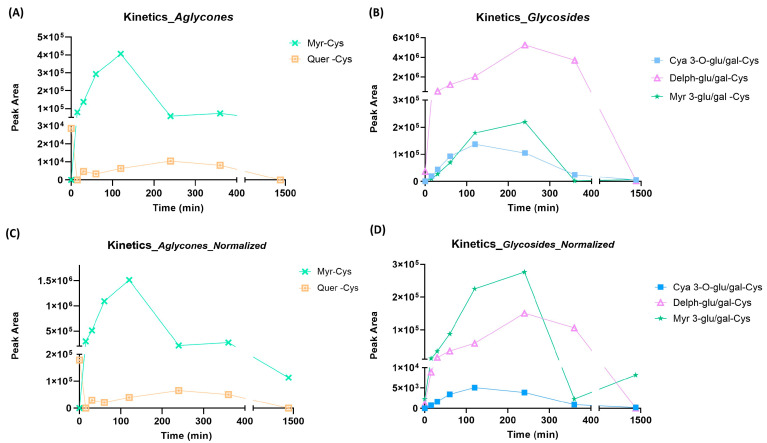
Peak area values as a function of time for the aglycones present in BPE (left panels, **A**,**C**) and the glycosidic forms (right panels, **B**,**D**); (panels **C**,**D**) correspond to normalized trends. Normalization considers the relative abundance of each ion; more specifically, the AUC was divided for the relative abundance.

It should be pointed out that the assumptions formulated so far are commensurate with the relative content of the individual species in the extract; therefore, it would be worth normalizing the AUC values calculated on the basis of the content of the starting flavonoids in order to have some indication of the reactivity of the individual molecules regardless of their distribution in the matrix (Figure 4).

The relative content of the adducts, normalized on the basis of the relative content of the individual components in the matrix (more specifically, the AUC was divided for the relative abundance), suggests a more pronounced reactivity of myricetin, both as aglycone and as a glycosidic derivative.

### 2.2. Protein Structure Analysis

#### 2.2.1. Targeted Protein Structure Analysis: Characterization of Potential Covalent Binders of M^pro^ by nLC-HR-MS/MS Analysis of the Incubation Mixture with BPE

**Figure 5:**
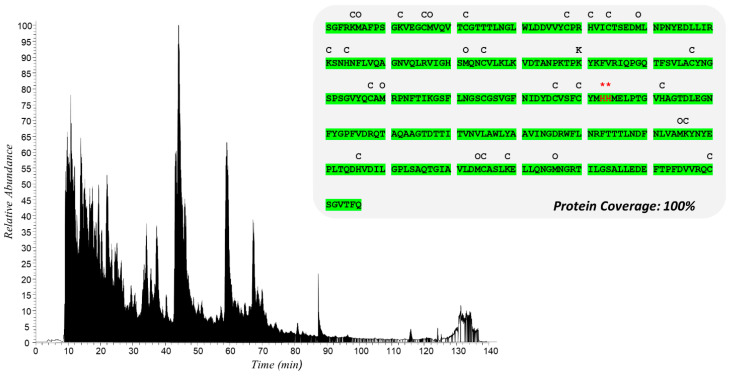
Total ionic current (TIC) of recombinant M^pro^ incubated with BPE and sequentially digested with trypsin and chymotrypsin. Above is shown the M^pro^ sequence with the peptide portions identified by nLC-HR-MS/MS analysis highlighted in green. Symbols above the sequence indicate modifications detected as follows: C, carbamidomethylation; O, oxidation; * delphinidin-3-glucoside/galactoside Micheal adduct on His163 or His164.

Figure 5 shows the chromatogram recorded during the nLC-HR-MS/MS analysis of the peptide mixture obtained by digesting with trypsin and chymotrypsin an aliquot of the BPE:M^pro^ (recombinant) incubation mixture. The ion trace is characterized by the presence of numerous peaks, ascribable to the elution of peptides whose sequence was then defined by in silico processing of the corresponding fragmentation spectra. Firstly, data processing confirmed the great potential of the S-TRAP™ spin columns, specifically designed to maximize the proteolytic digestion yield: the analysis led to an almost complete characterization of the primary sequence of M^pro^, with a protein coverage value close to 100% (Figure 5).

With the intent of following the kinetics of covalent adduct formation over time on the key residues of the active site of M^pro^, the MS spectra acquired were processed in a targeted mode, i.e., by selectively ‘tracing’ on the nucleophilic residues of M^pro^ only the mass shift (Δm) values noted during the metabolomic analysis, which can be ascribed to the formation of the most representative flavonoid adducts. The matching tasks automatically performed by the Proteome Discoverer software (version 2.2.0.338) provided a list of PSMs grouped by sequence homology, including both unmodified sequences and peptide sequences bearing plausible covalent adducts. For each peptide match, the best experimental fragmentation spectrum was also manually selected according to its concordance with the theoretical fragmentation spectrum simulated in silico: thus, a peptide modification can be considered confirmed only when the fragmentation spectra show b and/or y fragments adjacent to the modified residue.

Overall, after a meticulous evaluation of the reports issued by the PD software, one stable conjugate involving a peptide sequence bearing two adjacent histidine residues (His163 and His164), which although not involved in catalytic activity plays a decisive role in stabilizing the substrate in the binding pocket, was identified quite clearly and unequivocally in the sample collected 12 h after the start of incubation. Unfortunately, the fragmentation spectra do not allow the discernment of the two histidine residues accurately, since being contiguous in the sequence they produce fragment ions that are difficult to be assigned (ambiguous); their involvement has been rationalized through molecular dynamics studies. In detail, the mass shift value found could be explained by the formation of adducts with the corresponding aglycone; however, since only glycosidic derivatives are present in the matrix, we can only assume that the MS^2^ spectra is the result of a partial fragmentation in the ion source (artefact/s). 

**Figure 6:**
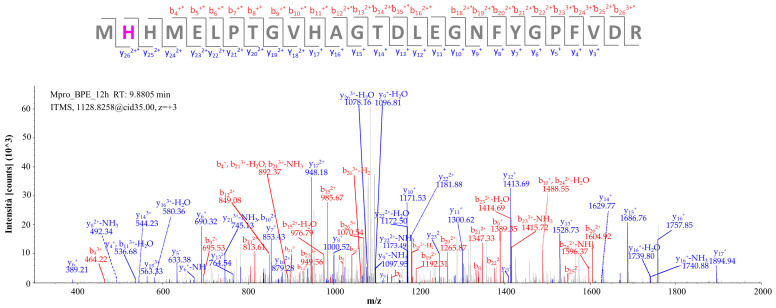
Fragmentation spectrum of the [M + 3H]^3+^ precursor ion at *m*/*z* 1128.15894 identified by computational analysis (* modified fragment ion); the corresponding theoretical fragmentation pattern, obtained by means of the software Proteome Discoverer, is reported in Table S1 (Supplementary Materials).

The fragmentation spectrum clarifying the identification of the modified peptide with sequence MH*HMELPTGVHAGTDLEGNFYGPFVDR carrying the delphinidin adduct on His163, indicated with an asterisk, is shown in Figure 6; precisely, the target residue is covalently conjugated to the aglycone portion of delphinidin (Michael adduct, which for simplicity we have called *Delph-agly_MA_R*). The fragmentation spectrum of the precursor ion [M + 3H]^3+^ having *m/z*: 1128.15894 (Figure 6) is characterized by almost all the fragments in the *y*-series and the *b*-series, confirming the assignment, with an XCorr value of 4.80.

#### 2.2.2. Target-Based Protein Structure Analysis of the Incubation Mixture of M^pro^ with Delphinidin-3-Glucoside Standard

The reactivity of the compound selected via targeted analysis was confirmed by incubating the recombinant protease with the standard of the glycoside derivative (delphinidin-3-glucoside). Similarly to the interpretation of experimental data collected for the incubation mixture with the extract, potential conjugated peptide/s was/were examined upon the incubation of M^pro^ with the standard at different times (2, 4, 12 h) and at two stoichiometric ratios, 1:1 and 1:2 (weight:weight). Overall data processing confirmed the formation of a Michael adduct under the following experimental incubation conditions: (i) 1:2 as stoichiometric ratio and (ii) after a 4 h time interval.

**Figure 7:**
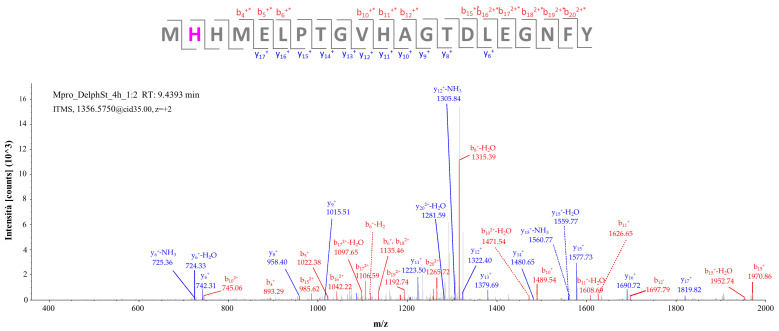
Fragmentation spectrum of the [M + 2H]^2+^ precursor ion at *m*/*z* 1356.56018 identified by computational analysis (* modified fragment ion); the corresponding theoretical fragmentation pattern obtained by means of the software Proteome Discoverer is reported in Table S2 (Supplementary Materials).

Figure 7 shows the fragmentation spectrum of the precursor ion [M + 2H]^2+^ having *m*/*z* 1356.56018 corresponding to the peptide MH*HMELPTGVHAGTDLEGNFY, in whose sequence the histidine residue marked with an asterisk represents the residue bearing the *Delph-agly_AM* adduct. The b-series and y-series fragments confirm the presence of the delphinidin aglycone modification on His163, with an XCorr value of 4.01.

Overall, targeted analysis strongly indicated a flavonoid compound in BPE capable of originating a stable conjugate (Michael adduct) with a histidine residue exposed in the binding pocket, i.e., His163 residue (or His164; it is not possible to identify the binding site with absolute certainty since they are proximal in the primary sequence); specifically, it is an anthocyanin in the form of glycosidic derivatives, the delphinidin-3-glucoside. Furthermore, it can be observed, based on the results obtained, that there is no time-dependent trend in the formation of the aforementioned adducts, but it is evident that the content is maximum at 12 h of incubation.

### 2.3. Evaluation of the Antiviral Activity

**Figure 8:**
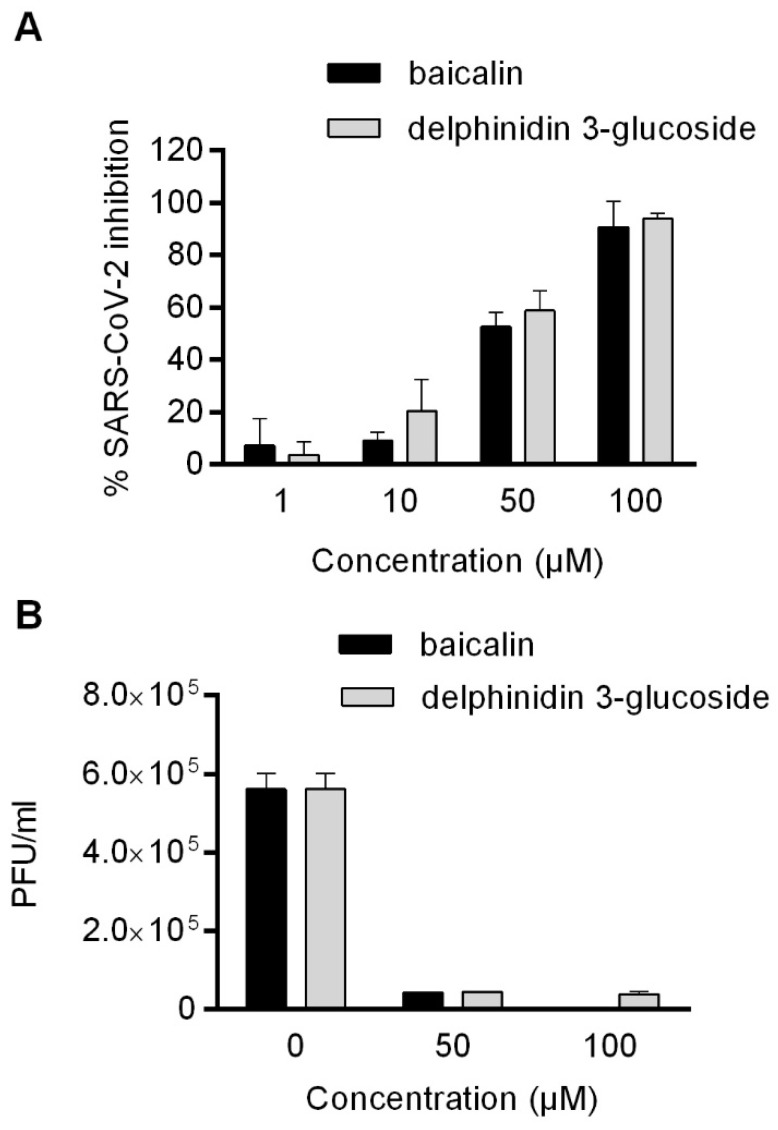
(**A**) Antiviral effect (expressed as % of inhibition) of baicalin and delphinidin-3-glucoside (μM) against SARS-CoV-2 measured by qRT-PCR assay. The inhibition of viral replication in Vero E6 cells by baicalin and delphinidin was expressed as the reduction of the viral load in the culture media. (**B**) Virucidal activity was confirmed by plaque reduction assays expressed as PFU/mL. Data were obtained from three independent experiments, and are expressed as mean ± SD.

Once the phytocomponents conjugated with the key residues in the binding pocket were identified, the antiviral activity of non-toxic concentrations was evaluated in Vero E6 cells exposed to a viral isolate purified from a SARS-CoV-2-infected patient. Viral replication was quantified in the culture media 48 h post-infection by q RT-PCR: a dose–response activity was observed for both baicalin and delphinidin-3-glucoside, as shown in Figure 8A. The IC_50_ values of baicalin and delphinidin were calculated and resulted in 52.45 ± 6.29 and 35.8 ± 1.38, respectively. To confirm these results, a plaque reduction assay was performed to test the potential inhibitory effect on virus infectivity of the phytocomponents. Both baicalin and delphinidin-3-glucoside showed the ability to completely reduce SARS-CoV-2 infectivity when used at a concentration ≥ 50 µM. 

### 2.4. Computational Studies

As described above, targeted analysis highlighted the formation of a stable Michael adduct between delphinidin-3-glucoside and one of the histidine residues constituting the M^pro^ pocket, His163 or His164. On this basis, computational studies were carried out in order to probe the engagement of these two residues in the binding of delphinidin-3-glucoside to M^pro^ active site. 

**Figure 9:**
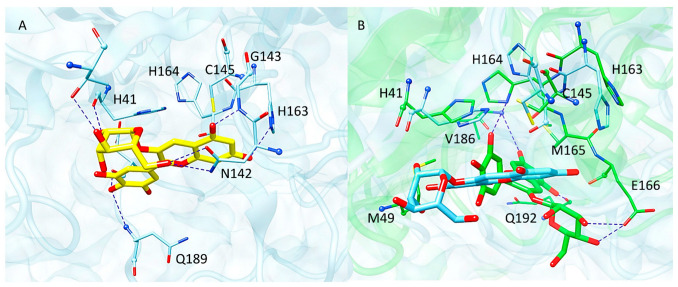
(**A**) Docking pose of delphinidin-3-glucoside (yellow sticks) within M^pro^ active site. (**B**) Representative structure of the MD simulation performed on delphinidin-3-glucoside–M^pro^ complex (green) superimposed to the starting coordinates obtained from docking (cyan). The residues of the binding pocket are displayed as sticks, while blue dashed lines represent H-bond interactions.

Molecular docking showed that this glycoside might interact with the catalytic pocket of M^pro^ by forming a network of H-bonds involving (i) the pyrogallol moiety and Asp187 and Gln189, (ii) the resorcinol hydroxyl groups and His163 and Gly143 and (iii) the sugar portion and Thr25, His41 and Asn 142 (Figure 9A). Moreover, the pyrogallol ring might establish a pi-stacking interaction with His41 while the benzopyrylium system is involved in hydrophobic and pi-sulfur contacts with the catalytic Cys145. While His163 is involved in the formation of a H-bond with the ligand, in the obtained docking pose its imidazole ring is distant from the electrophile sites of delphinidin-3-glucoside. Considering the high flexibility of M^pro^ active site [21], the interaction pattern of delphinidin-3-glucoside was further investigated by performing 750 ns of MD simulations by means of the software Amber18. The stability of the system was monitored by checking the RMSD profiles of both protein and ligand (Figure S2) which revealed that both entities reached stability in the second part of the trajectory, at about 450 ns, remaining stable for the rest of the simulation time. On this basis, cluster analysis was performed on this second part of the trajectory to obtain a representative conformation of the system. In Figure 9B, the representative structure of the most populated cluster superimposed to the starting docking complex is displayed. The outcomes showed that during the MD simulation, the glycoside moved from its starting position orienting the sugar moiety towards Glu166, which formed H-bonds with the hydroxyl groups of glucose. The benzopyrylium system moved away from Cys145, engaging Val186 and Gln192, which are involved in H-bonds with the resorcinol portion. Instead, the pyrogallol ring conveniently approached His164 and it was stabilized by (i) pi–pi interactions with His41, (ii) a H-bond with His164 and (iii) hydrophobic contacts with Met49. The distance between the Nδ of His164 and the electrophilic carbon at 2′ position of the pyrogallol moiety was recorded during the simulation time. As a result, values in the range of 3.37–12.55 Å were registered, suggesting that delphinidin-3-glucoside is able to adopt distances conducive to a Michael addiction in the appropriate conditions. Based on the obtained outcomes, our computational analysis supported the engagement of His164 in the formation of the covalent adduct. MM-GBSA calculations were carried out to estimate the free energy of binding ΔG of the protein–ligand complex. A ΔG value of −34.32 kcal/mol was obtained, indicating that the interaction between delphinidin-3-glucoside and M^pro^ is energetically favored.

## 3. Discussion

Since the outbreak, i.e., since January 2020, when WHO defined the SARS-CoV-2 epidemic as an international public health emergency, the scientific community has actively worked with the goal of countering the spread of the virus infection. Viruses, such as SARS-CoV-2, will always be present in human life being, thus finding new antiviral molecules is an important public health aspect. 

A molecule that effectively blocks the natural cleavage activity promoted by M^pro^ would be a compound with potential antiviral activity. 

Historically, natural products and their structural analogues provided important contributions to pharmacotherapy: plants with medicinal properties still represent not only a source of therapeutically bioactive molecules but also an advantageous starting point for the development of new drugs.

In this scenario, technological advances and improved analytical tools have greatly expanded the explorative power with respect to the possibility of searching chemical libraries with the sole aim of “repositioning” molecules whose efficacy has been already tested against other molecular targets [22,23]. 

Therefore, with the aim of expanding current knowledge, a complex analytical platform employing high-resolution mass spectrometry (HR-MS) and integrating the principles of metabolomics and protein structure analysis has been optimized to provide the opportunity to identify covalent binders of the M^pro^ protease active site, considering that the approach can be extended to other viruses proteases. The focus has been addressed on the cysteine-protease M^pro^ (main protease), given (i) its key role in mediating virus replication (the protease makes cleavages on the polyproteins leading, e.g., to the enzyme RNA polymerase release, which is essential for replication), (ii) its uniqueness, i.e., the fact that there are no proteins with a similar structure and function in humans, so that a compound that inhibits the viral protease activity would be less likely to have serious side effects on the patient, and finally (iii) its high conservation during the evolutionary process. A molecule that effectively blocks the natural cleavage activity promoted by M^pro^ would be a compound with potential antiviral activity.

In an early published paper, it was demonstrated how this methodological approach can achieve high levels of analytical performance in terms of sensitivity while operating in untargeted mode, thus enabling the complete characterization of covalent adducts between phytoconstituents and nucleophilic residues of M^pro^ without the need to make a priori assumptions [12]. The results obtained confirmed the formation of covalent adducts between the molecules used as a model of inhibition and three of the nucleophilic residues of M^pro^ exposed on the binding pocket: Cys145 and His41 constituting the catalytic dyad, and the vicinal histidine residues His163/His164.

With the intent of benefiting from the potential of the analytical approach just described and to begin to explore the panoply of matrices of natural origin, a mixture of phytoconstituents derived from blueberry, which is well-known as a rich source of anthocyanosides, was assayed. The choice of the extract was guided by the similarity of the functional groups characterizing its main components to those of baicalin and baicalein, thus suggesting potential reactivity toward the active site of the enzyme, as well as encouraging in silico data found in the literature that supported the selection of the matrix [13].

Overall, the results obtained led to the indisputable characterization of a covalent binder of the M^pro^ active site, namely the glycosidic derivative of delphinidin; listed below are some considerations about the relation between the structure of the molecule under investigation and its reactivity.

The first aspect to focus on is that delphinidin, quite similarly to baicalin (and corresponding aglycone baicalein), bears a pyrogallol portions, which is a moiety susceptible to nucleophilic attack. More specifically, the tri-hydroxylated ring of which the anthocyanoside is composed is, as such, unreactive; through a process of autoxidation, the corresponding oxidized intermediate is formed. The most likely oxidation product is the corresponding quinone, since other species, e.g., the semiquinone radical, have considerably shorter half-lives; quinones are more susceptible to nucleophilic attack by Michael addition or Schiff base formation by the side chains of target amino acid residues. We then hypothesize the pivotal role of the pyrogallol moiety in the formation of a stable conjugate with M^pro^, which effectively constitutes the chemically reactive group capable of creating covalent bonds with an amino acid at the catalytic site (warhead). The activity of flavonoids carrying a pyrogallol moiety has been reported by a recent paper by Xiao T. et al., who demonstrated that, in a panel of 15 natural compounds, only myricetin (the unique compound with a pyrogallol portion) inhibited M^pro^ at micromolar concentration [24]. We also observed a high reactivity of myricetin when the extract was incubated with cysteine (Figure 4), but considering the low abundance of myricetin in the extract we did not detect any adduct in the protein structure analysis. Another issue that should not be ignored is the susceptibility of the amino acid residues constituting the active site of cysteine protease to the formation of covalent adducts. Protein structure analysis has uniquely identified the formation of a stable conjugate involving the same protein moiety, namely the one bearing two histidine proximal residues: His163 and His164. These residues had already been identified as potential targets in the previous study and were confirmed to be susceptible to the binding of molecules possessing the chemical characteristics just described (catechol/pyrogallol moiety); we hypothesize that these aminoacidic residues, although not involved in protease catalytic activity, hold a key role in stabilizing the substrate in the binding pocket. It should be pointed out that, from the fragmentation spectra acquired, it is not possible to discern with absolute certainty the involvement of the two residues: being contiguous in the primary sequence, they produce fragment ions that are difficult to be assigned.

Last, but not least, we considered the kinetics of adduct formation: we had not observed a well-defined time-dependent trend, although the highest adduct content, estimated on the basis of the number of PSMs, is shown at the maximum incubation time tested, i.e., 12 h. It would be worthwhile to replicate the study by extending the incubation time to check the stability of the conjugates.

Molecular modelling studies were carried out in order to investigate the role of the histidine residues His163 and His164, identified as targets for the formation of the covalent adduct, in the protein–ligand binding mechanism. While molecular docking highlighted that delphinidin-3-glucoside might engage a H-bond with His163 through its resorcinol moiety, MD simulation revealed that this interaction is not stable throughout the trajectory. Indeed, during the MD, the ligand shifted from its original position conveniently approaching His164 through the pyrogallol ring which assumed distances suitable for a Michael addition. Therefore, in silico analysis suggested that His164 is more prone to forming the covalent adduct with the glycoside.

In light of what was previously described by Liu and colleagues [25], who showed the anti-SARS-CoV-2 activity of extracts from *S. baicalensis*, and baicalein in particular, we demonstrated that baicalin also acts against viral replication. The activity on the M^pro^ enzyme demonstrated by Liu and colleagues was 200 times higher for baicalein than baicalin, which is why they selected baicalein only for in vitro antiviral assays. In our model, baicalin inhibited SARS-CoV-2 replication by about 50% at 50 µM. Although it is not possible to directly compare results from different laboratories, the inhibition of SARS-CoV-2 replication by baicalin is only 15–20 times lower than that by baicalein; furthermore, we observed similar antiviral activity of delphinidin [13,26].

## 4. Materials and Methods

### 4.1. Reagents

Ultrapure water was prepared by a Milli-Q purification system (Millipore, Bedford, MA, USA). Delphinidin-3-*O*-glucoside chloride and baicalein-7-*O*-glucuronide were from Extrasynthese (Genay Cedex, France). Cysteine (Cys), iodoacetamide (IAA), tris(2-carboxyethyl)phosphine (TCEP) and tetraethylammonium bromide (TEAB), were provided by Sigma-Aldrich (Milan, Italy). Formic acid (FA), trifluoroacetic acid (TFA), acetonitrile (ACN) and all ultrapure (99.5%) grade solvents used in LC-MS analysis were also purchased from Sigma-Aldrich (Milan, Italy). S-TRAP™ columns were provided by Protifi (Huntington, NY, USA).

Vero E6 (ATCC CRL-1586™) clone cells were purchased from LGC Standards s.r.l. (Sesto San Giovanni, Italy), as the Italian distributor of ATCC (Manassas, VA, USA). Dulbecco’s Modified Eagle Medium (DMEM), fetal bovine serum (FBS), glutamine, penicillin and streptomycin were provided by EuroClone (Pero, Italy). For the qRT-PCR, a Nucleospin RNA virus kit was purchased from Macherey-Nagel (Duren, Germany) and Ag-Path one-step RT-PCR was purchased from Life Technologies (Carlsbad, CA, USA). Primers and probes for RT-PCR were purchased from Eurofins Genomics (Ebersberg, Germany). For the plaque reduction assay, formaldehyde was purchased from Applichem (Darmstadt, Germany). Methylene Blue was purchased from Merck (Darmstadt, Germany).

### 4.2. Plant Materials and Blueberry Extract Preparation

*Vaccinium corymbosum* L. cultivar Legacy berries selected for the project MIND FoodS Hub (Milano Innovation District Food System Hub, Milano, Italy) were frozen in liquid nitrogen, cold milled and stored at −80 °C. An extraction procedure was selected by evaluating the most common mixtures to extract polyphenols and in particular anthocyanins: methanol–water 0.1% HCOOH (80/20, % *v*/*v*) [27]. The ground powder was weighed in liquid nitrogen and then the extraction phase was added at a ratio of 2.5 mL/500 mg. 

The resulting suspension was vortexed for 30 s, sonicated for 30 min at room temperature and then centrifuged for 5 min at 5000 rpm. The supernatant was then separated from the pellet, the extraction was repeated twice and the supernatant was dried under vacuum and used for the analyses.

### 4.3. Metabolomics

#### 4.3.1. Analytical Profiling of the Blueberry Polyphenol Extract (BPE)

BPE was dissolved in EtOH:H_2_O (70/30, % *v*/*v*) and further diluted to a final concentration of 2 mg/mL in 0.1% formic acid and 0.5% DMSO. The analytical platform used comprises the Ultimate 3000 HPLC (Dionex, Sunnyvale, CA, USA), coupled to an LTQ Orbitrap XL mass spectrometer (Thermo Fisher Scientific, Waltham, MA, USA), set up as described by Baron et al. [28], to achieve the qualitative extract profiling and a further semi-quantitative evaluation. 

An in-house database for a targeted data analysis was built searching the literature for polyphenols contained in blueberry fruit, excepting those compounds that do not ionize (or only very poorly) in positive ion mode that were removed for convenience [29,30,31,32,33,34,35]. The database thus compiled contains 88 compounds, with their corresponding theoretical *m*/*z* value. The post-analysis search, within the acquired spectra, was performed by means of the Xcalibur QualBrowser tool (4.0, ThermoFisher Scientific Inc., Milan, Italy), using the exact mass (mass tolerance of 5 ppm), and manually checking the isotopic and the fragmentation patterns.

The relative percentage calculated for each compound (on the basis of the [M]^+^ or [M + H]^+^, considering that only monocharged ions were detected) as described by Equation (1) did in fact allow for only an estimation of their content in the mixture since it does not consider the different ionization efficiency of each molecule:

(1) $$\frac{{AUC}_{compound}}{\sum AUC}\times 100$$

#### 4.3.2. Electrophilic Compound Identification and Reaction Kinetics Study

A crucial step in this study was the identification of such components capable of promoting the formation of a Michael adduct with thiol groups, since a common feature of many M^pro^ inhibitors is the formation of a stable conjugate with the Cys145 residue constituting the catalytic dyad. The propensity of extract components to form covalent adducts was assessed by incubating the extract with free cysteine under physiological conditions. The experiment was set up so that different incubation times could be evaluated and a kind of reaction kinetics defined, even determining the time span in which the maximum adduct concentration is reached.

- Sample preparation

BPE: Cysteine

Cysteine was dissolved in 100 mM PBS pH 7.4 up to a final concentration of 1.25 mg/mL in the incubation mixture, while the BPE solution was prepared at 50 mg/mL in 8% DMSO 100 mM PBS pH 7.4 and further diluted up to a final concentration in the mixture of 6.25 mg/mL. Aliquots with a volume of 50 µL of the mixture were withdrawn and diluted 1:2 with H_2_O/CH_3_CN/HCOOH (70:30:0.1, % *v*/*v*), then 1:10 with H_2_O/HCOOH (100:0.1, % *v*/*v*) after 0, 2, 4, 6 and 24 h to stop the reaction. The stoichiometry of reaction, ~5:1 BPE:Cys (*w*/*w*), was chosen considering the high reactivity of cysteine.

- Reaction kinetic study by LC-HRMS (BPE: Cysteine)

The LC-HR-MS/MS method described in Section 4.3.1 was modified only in the chromatographic conditions setting to reduce the time of analysis to 32 min. Briefly, the multistep gradient was set as follows: 0–15 min from 1% of B to 20% of B, 15–25 min from 20% of B to 70% of B, 25–28 min isocratic of 70% B, 28.1–32 min isocratic of 1% B.

- MS data elaboration

Once the qualitative profile of BPE had been defined, the peculiar phytocomponent reactivity towards the cysteine’ thiol was speculated: Michael adduct structure formulae were assumed and the corresponding monoisotopic mass ([M]^+^ or [M + H]^+^) was calculated using Molecular Weight Calculator software (version 6.50). The ion current of each potential adduct was extracted from the chromatograms acquired as reported in Section 4.3.1, i.e., on the basis of the exact mass (mass tolerance of 5 ppm) and the isotopic pattern assumed.

### 4.4. Protein Structure Analysis

#### 4.4.1. Characterization and Localization of Protein Adducts

- M^pro^ incubation with BPE

Lyophilized recombinant M^pro^ was resuspended at a concentration of 1 µg/µL in 100 mM PBS pH 7.4, while the extract, based on its solubility, was dissolved in 8% DMSO 100 mM PBS pH 7.4 at a concentration of 120 µg/µL and further diluted in 100 mM PBS pH 7.4 up to a final concentration of 3 µg/µL, so as to reduce the relative content of DMSO to 0.2%. The incubation mixture Mpro:BPE was prepared at a stoichiometric ratio 1:3 (*w*/*w*), assuming slower kinetic than that obtained by incubating the extract with free cysteine, and was incubated in the Thermomixer at 37 °C, at a speed of 450 rpm, for 2, 4 and 12 h.

- Protein digestion (S-TRAP™ technology)

Samples collected at predefined withdrawal times were then processed according to the bottom-up protein structure analysis procedure reported by Baron et al. [12]. Given the negligible amount of recombinant protein incubated with the extract, in order to maximize the digestion yield, the great potential of the S-TRAP™ technology was exploited. The obtained peptide mixtures were dried in the SpeedVac (Martin Christ, Osterode, Germany) at 37 °C and stored at −80 °C until MS analysis.

- nLC-HR-MS/MS analysis (Orbitrap Elite™ Mass Spectrometer)

Peptide mixtures, resuspended in a volume of 0.1% TFA mobile phase, appropriate for three technical replicates (30 µL), were analyzed using a Dionex Ultimate 3000 nano-LC system (Sunnyvale, CA, USA) connected to an Orbitrap Elite™ Mass Spectrometer (Thermo Scientific, Brema, Germany) equipped with an ionization source, a Nanospray Ion Source (Thermo Scientific Inc., Milano, Italy), by applying the method previously set up and reported by Baron et al. [12].

- Targeted data analysis

Proteome Discoverer software (version 2.2.0.338, Thermo Fisher Scientific, Waltham, MA, USA), designed to computationally process full and tandem mass spectra, was used to handle raw data acquired by HRMS. The experimental mass spectra are matched against theoretical ones, the latter obtained by the in silico digestion of the M^pro^ sequence (Uniprot ID: P0DTD1, AA 3264-3569), by means of the SEQUEST algorithm, developed to automatically cross-validate the PSMs (peptide spectral matches) generated. 

For the targeted analysis, aimed at characterizing the protein adducts of *BPE* components with M^pro^, specific experimental parameters concerning the instrument setting for HRMS acquisition were listed in the processing workflow as already described by Baron et al. [12]. 

Furthermore, all the mass shifts considered plausible according to the hypothesized reaction mechanisms (Micheal addition) were also included as variable modifications targeting the nucleophilic moieties of Cys and His (as previously reported [12]), of which some structure formulae are reported in Figure S3 (Supplementary Materials).

#### 4.4.2. Characterization of Protein Adducts Deriving from the M^pro^ Incubation with Delphinidin-3-Glucoside

Based on preliminary investigations carried out on the extract as a whole, and the resulting findings on the reactivity of the individual components, it was decided to focus on the one molecule capable of forming a stable conjugate with the protease binding site residues, namely the glycosidic derivative of delphinidin. Given the solubility of the two standard molecules, solutions were prepared in 100 mM PBS pH 7.4 at a concentration of 1 µg/µL. The stoichiometric ratio of incubation M^pro^:delphinidin-3-glucoside was chosen considering the relative abundance of the two molecules in the extract tested (1:1/1:2); the incubation mixture was kept at 37 °C in the Thermomixer, setting a shaking speed of 450 rpm, for 2, 4 and 12 h. Aliquots withdrawn from the incubation mixture at the predefined timepoints were processed according to the experimental protocol reported in Section 4.4.1, point Protein digestion (S-TRAP™ technology). The collected peptide mixtures were dried and stored at −80 °C until MS analysis performed as already described in point nLC-HR-MS/MS analysis (Orbitrap Elite™ Mass Spectrometer). Also, the raw data obtained at this stage were processed using the Proteome Discoverer software (version 2.2.0.338, Thermo Fisher Scientific, Waltham, MA, USA); the parameters are completely comparable to those reported in point Targeted data analysis, except for the set of variable modifications restricted to plausible adducts occurring in the presence of only delphinidin (Figure S3D, Supplementary Materials).

### 4.5. In Vitro Evaluation of Antiviral Activity

#### 4.5.1. Cell Culture and Virus

Vero E6 clone cells were maintained in culture with Dulbecco’s Modified Eagle Medium (DMEM) supplemented with 10% of foetal bovine serum, 2 mM glutamine, 100 U/mL penicillin, and 100 mg/mL streptomycin. SARS-CoV-2 viral strain B.1 (SARS-CoV-2/human/ITA/Milan-UNIMI-1/2020, Gen Bank accession ID: MT748758.1, GISAID accession: EPI_ISL 584051) was isolated from a COVID-19 patient’s nasopharyngeal swab and titrated by plaque assay, as described by Delbue et al. [36].

#### 4.5.2. Infection, Treatment with Baicalin and Delphinidin-3-Glucoside

Vero E6 cells were seeded into 96-well plates at a density of 1.5 × 10^4^ cells/well and incubated for 24 h at 37 °C, 5% CO_2_. Cells (approximately 3 × 10^4^ cells/well) were treated with baicalin (used as positive control) and delphinidin-3-glucoside (100–50−10–1 µM) for 1 h at 37 °C, 5% CO_2_ (cells pre-treatment). In parallel, the virus (MOI 0.05) was incubated for 1 h at 37 °C in a 96-well plate in the presence of the same doses of the compounds, in a volume of 50 µL (virus pre-treatment). Following the pretreatments, the compounds were removed from the cells and 30 µL of the virus–compound mixture was inoculated on the cells, for 2 h at 37 °C, 5% CO_2_. After virus adsorption and viral inoculum removal, new medium with fresh compounds was added and kept for further 48 h. Infected, untreated cells were used as control of infection. 

#### 4.5.3. Antiviral Assays

SARS-CoV-2 replication was evaluated in cell media 48 h post infection. Ten microliters of supernatants from treated or untreated (controls) wells were mixed with 10 μL PBS and inactivated at 98° for 10 min. Specific qRT-PCR, targeting the viral N1 gene, was performed [37]. Results were expressed as % inhibition of SARS-CoV-2 replication, calculated according to the following formula: 100- % SARS-CoV-2 replication, where SARS-CoV-2 replication = 100 × (SARS-CoV-2 copies/mL treated sample/SARS-CoV-2 copies/mL untreated control).

#### 4.5.4. Evaluation of Virucide Activity by Plaque Reduction Assay

Virucide activity of baicalin and delphinidin-3-glucoside was evaluated by plaque reduction assay on Vero E6 cells, as previously described [38]. Briefly, Vero E6 cells were seeded in 6-well plates (approximately 4 × 10^5^ cells/well) for 24 h at 37 °C, 5% CO_2_. Different ten-fold dilutions of supernatants from antiviral assays plates (see paragraph 4.5.3) were added to the wells for 2 h at 37 °C, 5% CO_2_. Subsequently, virus inoculum was removed, and cells were covered with 0.3% agarose dissolved in cell medium at 37 °C for 48 h, 5% CO_2_. Cells were fixed with 4% formaldehyde solution and, after agarose removal, stained with methylene blue. Results were expressed as Plaque Forming Unit (PFU)/mL, calculated considering the dilution factor and the inoculum volume, and as a percentage of virus inhibition, compared to untreated infected cells.

#### 4.5.5. Statistical Analysis

Statistical differences were analyzed via Student’s *t*-test and a value of *p* ≤ 0.05 was considered significant.

### 4.6. Computational Studies

#### 4.6.1. Molecular Docking

The binding mode of delphinidin-3-glucoside into the SARS-CoV-2 M^pro^ active site was clarified by molecular docking employing the crystal structure of M^pro^ in complex with baicalein (PDB ID 6M2N) [39]. The protein was prepared as described elsewhere [40]. The 3D structure of delphinidin-3-glucoside was retrieved from PubChem [41] and optimized by the PM7 semi-empirical method [42]. Docking simulation was carried out by means of PLANTS software (v 1.2) [43] following the protocol reported by Vittorio et al. [40] with minor modifications. Specifically, the binding pocket was defined in order to include the residues within 10 Å from the co-crystallized ligand while the number of cluster structures was set to 10. ChemPLP and speed1 were selected as scoring function and search speed, respectively. The docking protocol was validated by re-docking the native ligand resulting in the successful reproduction of the experimentally observed binding mode with a RMSD value of 1.87 Å. The lowest scored pose was selected for the following studies. 

#### 4.6.2. Molecular Dynamics Simulation

Molecular dynamics simulation (MD) was performed by means of Amber 18 package [44] using the complex obtained from the above described docking procedure as starting coordinates. The parametrization of the system and the protocol used to set up and run the simulation are reported in our previous paper [12]. A production run of 750 ns was carried out in NPT ensemble without any restraint. The cpptraj module [45] of AmberTools 18 was used for the RMSD calculation. Cluster analysis was performed by means of TTClust tool (version 4.10.3) [46], considering only the last part of the trajectory (from 450 ns to the end of the trajectory) as the system reached stability in this period.

## 5. Conclusions

Overall, the platform enabled the identification, with good certainty, of a flavonoid compound contained in the blueberry polyphenol extract capable of forming stable Michael adducts with a histidine residue (His163/164) exposed in the M^pro^ cysteine-protease binding pocket. The described method, in addition to enabling the fishing out of covalent binders as potential inhibitors of M^pro^, allowed the formulation of hypotheses about the mechanism of action of the inhibitor. The great efficiency of the developed platform is coupled to its great applicability: starting from the same experimental workflow, in fact, different matrices of natural origin can be examined in the future with the aim of expanding the current know-how regarding molecules with antiviral activity effective against SARS-CoV-2.

## Figures and Tables

**Table 1:** Summary of information on the compounds found in BPE: Peak Number; compound: annotations from chemical libraries of natural compounds; chemical formula; [M]^+^/[M + H]^+^ calculated: monoisotopic mass calculated on the basis of the chemical formulae (theoretical M_mi_); experimental [M]^+^/[M + H]^+^: experimental value of monoisotopic mass; Δ ppm: calculated accuracy value [(M_mi_ exp. − M_mi_ th.)/Mmi th. × 10^6^]; RT (min): retention time; MS/MS: characteristic fragment ions; Peak Area (AUC); Relative Abundance (%): percentage relative abundance value.

Peak N°	Compound	Chemical Formula	Th. [M]^+^/[M + H]^+^	Exp. [M]^+^/[M + H]^+^	Δ ppm	MS/MS	RT (min)	Rel. Ab. (%)
**1**	Delphinidin-3-glucoside/galactoside	C_21_H_21_O_12_^+^	465.1033	465.10201	−2.77	303	3.33	12.11
**2**	Delphinidin-3-arabinoside	C_20_H_19_O_11_^+^	435.09273	435.09188	−1.95	303	4.31	6.14
**3**	Cyanidin-3-glucoside/galactoside	C_21_H_21_O_11_^+^	449.10838	449.1074	−2.18	287	4.59	5.33
**4**	Petunidin-3-glucoside/galactoside	C_22_H_23_O_12_^+^	479.11895	479.11793	−2.13	317	5.2	12.72
**5**	Cyanidin-3-arabinoside	C_20_H_19_O_10_^+^	419.09782	419.09731	−1.22	287	6.58	2.87
**6**	Petunidin-3-arabinoside	C_21_H_21_O_11_^+^	449.10838	449.10775	−1.4	317	7.5	5.83
**7**	Peonidin-3-glucoside/galactoside	C_22_H_23_O_11_^+^	463.12403	463.12319	−1.81	301	7.5	1.4
**8**	Malvidin-3-glucoside/galactoside	C_23_H_25_O_12_^+^	493.1346	493.13362	−1.99	331	8.67	22.99
**9**	Peonidin-3-arabinoside	C_21_H_21_O_10_^+^	433.11347	433.11285	−1.43	301	10.43	0.82
**10**	Malvidin-3-arabinoside	C_22_H_23_O_11_^+^	463.12403	463.12326	−1.66	331	11.57	14.2
**11**	Myricetin-3-glucoside/galactoside	C_21_H_21_O_13_	481.09821	481.0973	−1.9	319	13.84	1.1
**12**	Quercetin-3-glucoside/galactoside	C_21_H_21_O_12_	465.1033	465.10255	−1.61	303	20.96	8.64
**13**	Quercetin-3-glucuronide	C_21_H_19_O_13_	479.08256	479.08165	−1.9	303	21.53	0.53
**14**	Quercetin-3-arabinoside/xyloside	C_20_H_19_O_11_	435.09273	435.09216	−1.32	303	25.92	3.42
**15**	Quercetin-3-rhamnoside	C_21_H_21_O_11_	449.10838	449.10793	−1.01	303	29.77	1.59
**16**	Isorhamnetin-3-glucoside/galactoside	C_22_H_23_O_12_	479.11895	479.11824	−1.47	317	30.32	0.29
**17**	Myricetin	C_15_H_11_O_8_	319.04539	319.04512	−0.85	181	31.04	0.02
**18**	Quercetin	C_15_H_11_O_7_	303.05048	303.05008	−1.31	153–181	47.85	0.02

**Table 2:** Molecular formulae and calculated monoisotopic masses of the hypothesized Michael adducts with Cys.

Compound	Chemical Formula	Th. [M]^+^/[M + H]^+^
Cyanidin-3-arabinoside	C_23_H_24_NO_12_S^+^	538.10192
Cyanidin-3-glucoside/galactoside	C_24_H_26_NO_13_S^+^	568.11248
Delphinidin-3-arabinoside	C_23_H_24_NO_13_S^+^	554.09683
Delphinidin-3-glucoside/galactoside	C_24_H_26_NO_14_S^+^	584.10740
Myricetin	C_18_H_15_NO_12_S	470.03932
Myricetin-3-glucoside/galactoside	C_24_H_25_NO_15_S	600.10231
Petunidin-3-arabinoside	C_24_H_26_NO_13_S^+^	568.11248
Petunidin-3-glucoside/galactoside	C_25_H_28_NO_14_S^+^	598.12304
Quercetin-3-arabinoside/xyloside	C_23_H_23_NO_13_S	554.09683
Quercetin-3-glucoside/galactoside	C_25_H_27_NO_13_S	582.12813
Quercetin-3-rhamnoside	C_24_H_25_NO_13_S	568.11248
Quercetin	C_18_H_15_NO_9_S	422.05457
Quercetin-3-glucuronide	C_24_H_23_NO_15_S	598.08666

## Data Availability

The data presented in this study are available on request from the corresponding author.

## Supplementary Materials

The following supporting information can be downloaded at: https://www.mdpi.com/article/10.3390/molecules29112702/s1, Figure S1: Extracted ionic current (XIC) for the 18 compounds identified in BPE by LC-HRMS and numbered according to the progressive elution as shown in Table 2; Table S1: Theoretical fragmentation pattern obtained for the [M + 3H]^3+^ precursor ion at *m*/*z* 1128.15894 identified by means of the software Proteome Discoverer (* modified fragment ion); Table S2: Theoretical fragmentation pattern obtained for the [M + 2H]^2+^ precursor ion at *m*/*z* 1356.56018 identified by means of the software Proteome Discoverer (* modified fragment ion); Figure S2: RMSD profiles of protein and ligand recorded during the MD simulation of delphinidin-3-glucoside–M^pro^ complex; Figure S3: Structure formulae of the modifications to be investigated that target the nucleophilic residues of M^pro^, divided into sections (A–E) on the basis of the starting phytocomponent.

## List of Abbreviation

ACN	Acetonitrile
AUC	Area under the curve
BPE	Blueberry polyphenol extract
Cys	Cysteine
DMEM	Dulbecco’s Modified Eagle Medium
FA	Formic acid
FBS	Fetal bovine serum
His	Histidine
HR-MS	High-resolution mass spectrometry
HTS	High Throughput Screening
IAA	Iodoacetamide
LC-HRMS	Liquid Chromatography High Resolution Mass Spectrometry
MD	Molecular dynamics
MS	Mass Spectrometry
M^pro^	Main protease
Nsp	Non-structural proteins
PD	Proteome Discoverer
PSMs	Peptide spectral matches
TCEP	Tris(2-carboxyethyl)phosphine
TEAB	Tetraethylammonium bromide
TFA	Trifluoroacetic acid
TIC	Total ionic current
