# Supplementary material for: Screening of Mpro Protease (SARS-CoV-2) Covalent Inhibitors from an Anthocyanin-Rich Blueberry Extract Using an HRMS-Based Analytical Platform
Source: Molecules. 2024 Jun 6;29(11):2702. doi: 10.3390/molecules29112702 (PMC11173886; doi:10.3390/molecules29112702)
Supplement: Supplementary file 1 [file molecules-29-02702-s001.zip › molecules-3017736-supplementary.pdf]

Supplementary Material

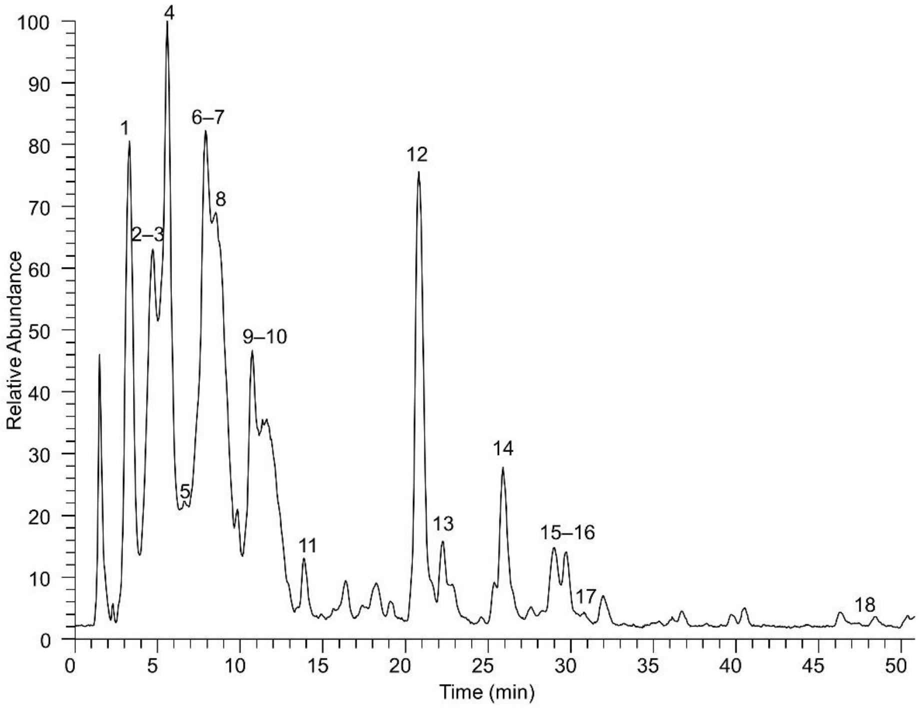

Figura S1. Extracted ionic current (XIC) for the 18 compounds identified in BPE by LC-HRMS and numbered according to the progressive elution as shown in **Table 2**.

#1	b^+	b^{2+}	b^{3+}	Sequence	y^+	y^{2+}	y^{3+}	#2
1	132.04776	66.52752	44.68744	M				27
2	567.11802	284.06265	189.71086	**H-*Delph-agly_MA_R***	3251.37995	1626.19361	1084.46483	26
3	761.19840	381.10284	254.40432	H-***Carbamidomethylation***	2816.30969	1408.65848	939.44141	25
4	892.23888	446.62308	298.08448	M	2622.22931	1311.61829	874.74796	24
5	1021.28148	511.14438	341.09868	E	2491.18883	1246.09805	831.06779	23
6	1134.36554	567.68641	378.79336	L	2362.14624	1181.57676	788.05360	22
7	1231.41830	616.21279	411.14429	P	2249.06217	1125.03472	750.35891	21
8	1332.46598	666.73663	444.82685	T	2152.00941	1076.50834	718.00799	20
9	1389.48745	695.24736	463.83400	G	2050.96173	1025.98450	684.32543	19
10	1488.55586	744.78157	496.85680	V	1993.94027	997.47377	665.31827	18
11	1625.61477	813.31102	542.54311	H	1894.87185	947.93956	632.29547	17
12	1696.65189	848.82958	566.22215	A	1757.81294	879.41011	586.60916	16
13	1753.67335	877.34031	585.22930	G	1686.77583	843.89155	562.93013	15
14	1854.72103	927.86415	618.91186	T	1629.75436	815.38082	543.92297	14
15	1969.74797	985.37762	657.25417	D	1528.70668	764.85698	510.24041	13
16	2082.83203	1041.91966	694.94886	L	1413.67974	707.34351	471.89810	12
17	2211.87463	1106.44095	737.96306	E	1300.59568	650.80148	434.20341	11
18	2268.89609	1134.95168	756.97021	G	1171.55308	586.28018	391.18921	10
19	2382.93902	1191.97315	794.98452	N	1114.53162	557.76945	372.18206	9
20	2530.00743	1265.50735	844.00733	F	1000.48869	500.74798	334.16775	8
21	2693.07076	1347.03902	898.36177	Y	853.42028	427.21378	285.14494	7
22	2750.09222	1375.54975	917.36893	G	690.35695	345.68211	230.79050	6
23	2847.14499	1424.07613	949.71985	P	633.33549	317.17138	211.78335	5
24	2994.21340	1497.61034	998.74265	F	536.28272	268.64500	179.43243	4
25	3093.28182	1547.14455	1031.76546	V	389.21431	195.11079	130.40962	3
26	3208.30876	1604.65802	1070.10777	D	290.14590	145.57659	97.38682	2
27				R	175.11895	88.06311	59.04450	1

Table S1. Theoretical fragmentation pattern obtained for the $[M+3H]^{3+}$ precursor ion at m/z 1128.15894 identified by means of the software *Proteome Discoverer* (*modified fragment ion).

#1	b^+	b^{2+}	Sequence	y^+	y^{2+}	#2
1	132.04776	66.52752	M			21
2	568.12585	284.56656	**H-*Delph-agly_MA***	2581.04867	1291.02797	20
3	762.20623	381.60675	H-***Carbamidomethylation***	2144.97058	1072.98893	19
4	893.24671	447.12700	M	1950.89020	975.94874	18
5	1022.28931	511.64829	E	1819.84972	910.42850	17
6	1135.37337	568.19032	L	1690,80713	845.90720	16
7	1232.42613	616.71671	P	1577.72306	789.36517	15
8	1333.47381	667.24054	T	1480.67030	740.83879	14
9	1390.49528	695.75128	G	1379.62262	690.31495	13
10	1489.56369	745.28548	V	1322.60116	661.80422	12
11	1626.62260	813.81494	H	1223.53274	612.27001	11
12	1697.65972	849.33350	A	1086.47383	543.74055	10
13	1754.68118	877.84423	G	1015.43672	508.22200	9
14	1855.72886	928.36807	T	958.41525	479.71126	8
15	1970.75580	985.88154	D	857.36757	429.18743	7
16	2083.83986	1042.42357	L	742.34063	371.67395	6
17	2212.88246	1106.94487	E	629.25657	315.13192	5
18	2269.90392	1135.45560	G	500.21397	250.61063	4
19	2383.94685	1192.47706	N	443.19251	222.09989	3
20	2531.01526	1266.01127	F	329.14958	165.07843	2
21			Y	182.08117	91.54422	1

Table S2. Theoretical fragmentation pattern obtained for the $[M+2H]^{2+}$ precursor ion at m/z 1356.56018 identified by means of the software *Proteome Discoverer* (*modified fragment ion).

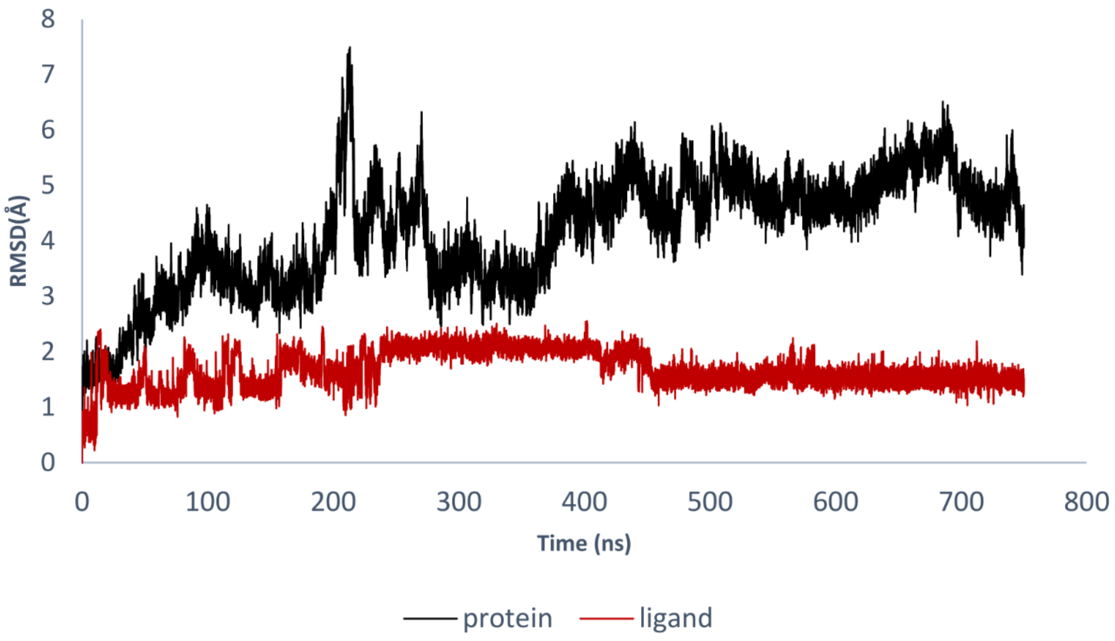

Figure S2. RMSD profiles of protein and ligand recorded during the MD simulation of delphinidin 3-glucoside- M^{pro} complex.

(A)

Cyanidin / Cyanidin-glycoside

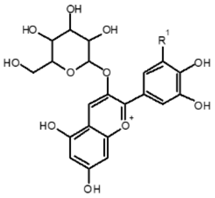

Cyanidin 3 glucoside/galactoside Michael Adduct (Cya-3-glu/gal_MA)
R^1: S-Cys; N-His

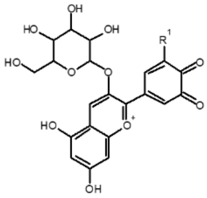

Cyanidin 3 glucoside/galactoside Michael Adduct *rearrangement* (Cya-3-glu/gal_MA_R)
R^1: S-Cys; N-His

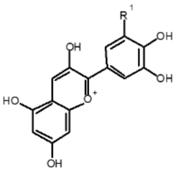

Cyanidin aglycon Michael Adduct (Cya-agly_MA)
R^1: S-Cys; N-His

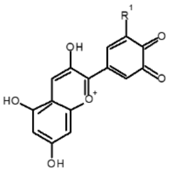

Cyanidin aglycon Michael Adduct *rearrangement* (Cya-agly_MA)
R^1: S-Cys; N-His

(B)

Petunidin / Petunidin-glycoside

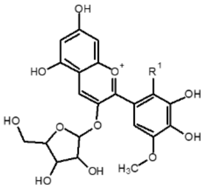

Petunidin 3 arabinoside Michael Adduct (Pet-3-ara_MA)
R^1: S-Cys; N-His

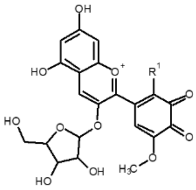

Petunidin 3 arabinoside Michael Adduct *rearrangement* (Pet-3-ara_MA_R)
R^1: S-Cys; N-His

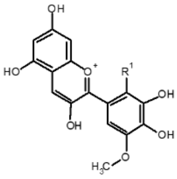

Petunidin aglycon Michael Adduct (Pet-agly_MA)
R^1: S-Cys; N-His

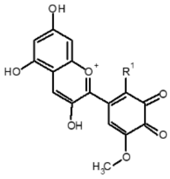

Petunidin aglycon Michael Adduct *rearrangement* (Pet-agly_MA_R)
R^1: S-Cys; N-His

(C)

Quercetin aglycon

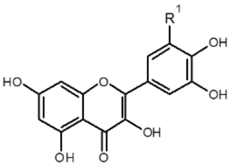

Quercetin aglycon Michael Adduct (Quer-agly_MA)
R^1: S-Cys; N-His

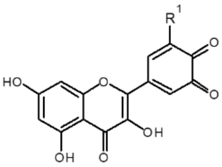

Quercetin aglycon Michael Adduct *rearrangement* (Quer-agly_MA_R)
R^1: S-Cys; N-His

(D)

Delphinidin / Delphinidin-glycoside

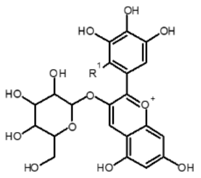

Delphinidin 3 glucoside/galactoside Michael Adduct (Delph-3-glu/gal_MA)
R[1]: S-Cys; N-His

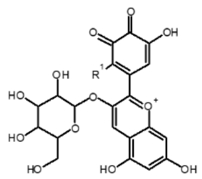

Delphinidin 3 glucoside/galactoside Michael Adduct *rearrangement* (Delph-3-glu/gal_MA_R)
R[1]: S-Cys; N-His

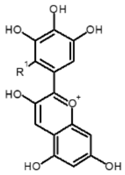

Delphinidin aglycon Michael Adduct (Delph-agly_MA)
R[1]: S-Cys; N-His

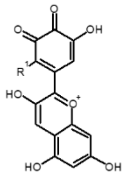

Delphinidin aglycon Michael Adduct *rearrangement* (Delph-agly_MA_R)
R[1]: S-Cys; N-His

(E)

Myricetin / Myricetin-glycoside

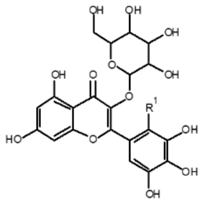

Myricetin 3 glucoside/galactoside Michael Adduct (Myr-3-glu/gal_MA)
R[1]: S-Cys; N-His

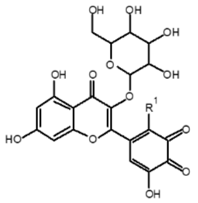

Myricetin 3 glucoside/galactoside Michael Adduct *rearrangement* (Myr-3-glu/gal_MA_R)
R[1]: S-Cys; N-His

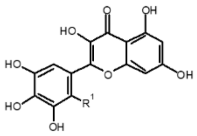

Myricetin aglycon Michael Adduct (Myr-agly_MA)
R[1]: S-Cys; N-His

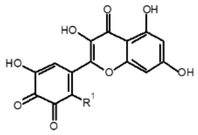

Myricetin aglycon Michael Adduct *rearrangement* (Myr-agly_MA_R)
R[1]: S-Cys; N-His

Figure S3. Structure formulae of the modifications to be investigated that target the nucleophilic residues of M^{pro}, divided into sections (A-E) on the basis of the starting phytocomponent.
